# Genetic and Epigenetic Changes during the Upward Expansion of *Deyeuxia angustifolia* Kom. in the Alpine Tundra of the Changbai Mountains, China

**DOI:** 10.3390/plants10020291

**Published:** 2021-02-03

**Authors:** Biao Ni, Jian You, Jiangnan Li, Yingda Du, Wei Zhao, Xia Chen

**Affiliations:** 1 National & Local United Engineering Laboratory for Chinese Herbal Medicine Breeding and Cultivation, School of Life Sciences, Jilin University, Changchun 130012, China; nibiao1991@163.com (B.N.); jianyou@jlu.edu.cn (J.Y.); lijiangnan@jlu.edn.cn (J.L.); 2 School of Life Sciences, Jilin University, Changchun 130012, China; duyingda@jlu.edu.cn

**Keywords:** *Deyeuxia angustifolia* Kom., upward expansion, genetic, epigenetic, adaptation

## Abstract

Ecological adaptation plays an important role in the process of plant expansion, and genetics and epigenetics are important in the process of plant adaptation. In this study, genetic and epigenetic analyses and soil properties were performed on *D. angustifolia* of 17 populations, which were selected in the tundra zone on the western slope of the Changbai Mountains. Our results showed that the levels of genetic and epigenetic diversity of *D. angustifolia* were relatively low, and the main variation occurred among different populations (amplified fragment length polymorphism (AFLP): 95%, methylation sensitive amplification polymorphism (MSAP): 87%). In addition, DNA methylation levels varied from 23.36% to 35.70%. Principal component analysis (PCA) results showed that soil properties of different populations were heterogeneous. Correlation analyses showed that soil moisture, pH and total nitrogen were significantly correlated with genetic diversity of *D. angustifolia*, and soil temperature and pH were closely related to epigenetic diversity. Simple Mantel tests and partial Mantel tests showed that genetic variation significantly correlated with habitat or geographical distance. However, the correlation between epigenetic variation and habitat or geographical distance was not significant. Our results showed that, in the case of low genetic variation and genetic diversity, epigenetic variation and DNA methylation may provide a basis for the adaptation of *D. angustifolia.*

## 1. Introduction

Global warming has many impacts on plants. In particular, a warmer climate may force plant species to migrate from low to high altitudes [1], which is a phenomenon that has been recently documented in some Chinese mountains and other parts of the world [2,3,4,5,6]. The rapid phenotypic changes of adaptive traits provide organisms with the potential for local adaptation, which is very important for the survival and expansion of organisms [7,8]. Studies have shown that plants adapt to different environments in many ways, and genomic variation provides the basis for plant adaptation and invasiveness [9,10,11]; the large expansion of plant transposons can produce genetic variation and lead to variations in key adaptive traits, thereby improving their adaptability [12]. Plants adapt to the environment via natural selection of fixed traits and plasticity of variable traits [13,14,15].

Studies shown that some invasive plants have similar or higher genetic diversity in the invaded region than in their original habitat, which may help them adapt to the environment [16,17]. Studies on the genetic structure of the invasive plant *Phyla canescens* showed that the invasive populations in Australia and France had similar or even higher genetic diversity than those in their native areas [18]. Generally, species need to maintain enough genetic variation, that is, rich genetic diversity, to adapt to the environment and cope with natural selection. For invasive species, enough genetic variation is conducive to coping with the selection pressure in a new environment. Although environmental adaptation is accomplished by genetic changes of mutation, drift and selection are very slow for some introduced species, and epigenetic mechanisms can affect their ecological phenotypic diversity through rapid gene expression regulation [19,20], thereby affecting their ecological performance, evolutionary potential and successful invasion [15,21,22]. As a mechanism of adaptive plasticity of natural plant populations, epigenetic variation may be an important factor of the successful invasion of plants in a wide range of environments [23]. Among all known epigenetic mechanisms, DNA methylation is relatively stable, as it has intergenerational heritability and can be independent of heritable genes [24,25,26]. Environmental stresses might trigger DNA methylation; studies have shown that DNA methylation plays an important role in plants’ stress tolerance, such as water deficits, chromium and high-salinity soils [27,28,29,30]. Thus, the study of ecology and evolutionary epigenetics has attracted the most attention, and it is widely used in the study of epigenetic variation in invasive plants [13,31,32].

The gramineous plant *Deyeuxia angustifolia* (Komarov) Y. L. Chang (*D. angustifolia*), originally distributed on the low altitude birch forest belt of the Changbai Mountains, is one of the dominant herbs under birch forest. However, field observations in the past 30 years have shown that after growth and reproduction, *D. angustifolia* at lower elevations of the tundra have been connected into patches. The increasing biomass of *D. angustifolia* has been able to exclude native plants such as *Rhododendron chrysanthum Pall.* and *Vaccinium uliginosum Linn.* and became the only dominant species [33,34]. The main reason for the upward expansion of the tundra belt on *D. angustifolia* was that a large number of forest gaps were formed due to the typhoon that occurred in 1986, which created a natural expansion opportunity for low altitude plants [35]. In the subsequent warming and nitrogen deposition experiments, the researchers compared the effects of warming and nitrogen deposition on the expansion of the *D. angustifolia* plant and the native *Rhododendron chrysanthum Pall.* plant in the tundra of the Changbai Mountains. The results showed that nitrogen deposition significantly promoted the growth of *D. angustifolia*. Nutrient disturbance may be the main reason for the expansion of *D. angustifolia* into tundra ecosystem of the Changbai Mountains [36]. The previous studies of our research group showed that the expansion of *D. angustifolia* significantly reduced the species diversity and aboveground biomass of the native plant community, and changed the soil microbial community structure of native plants in the tundra belt [37]. Consequently, previous studies mainly explored the reasons for the expansion of *D. angustifolia* into the tundra zone and its impact on native plants in the tundra. However, the adaptation mechanism of *D. angustifolia* invading the tundra is still unclear. In the end, we will gain better knowledge on how *D. angustifolia*, and probably other invasive species, adapt to stressful environments.

In this study, we sampled 17 natural populations of *D. angustifolia* from different elevations on the western slope of the Changbai Mountains and surveyed the environmental factors of each sampling site in order to detect different environment conditions. Amplified fragment length polymorphism (AFLP) and methylation sensitive amplification polymorphism (MSAP) were used to study the genetic and epigenetic variation of *D. angustifolia* and to unravel the role of genetic and epigenetic variation in the adaptation of *D. angustifolia*.

## 2. Results

### 2.1. Genetic Diversity and Structure

**Figure 1:**
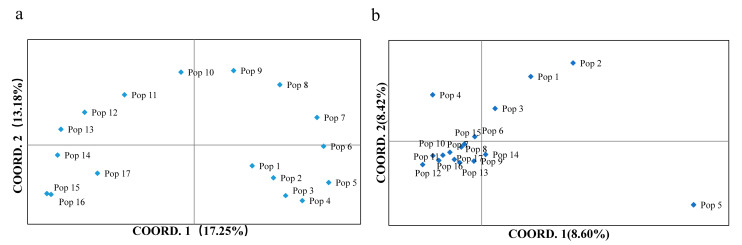
Principal coordinates analyses (PCoA) of Nei’s genetic distance (**a**) and Nei’s epigenetic distance (**b**) of different populations of *D. angustifolia.*

Using AFLP molecular marker technology, 10 pairs of selective amplification primers were used to analyze the genetic diversity and genetic structure of 17 natural *D. angustifolia* populations (Table 1). A total of 1776 loci were detected. The Shannon’s information index (I) for each population ranged from 0.009 (P08) to 0.164 (P17), with an average of 0.035; the percentage of polymorphic loci (PPL) ranged from 1.75% (P08) to 31.14% (P17) with an average of 6.79% (Table 2). The results showed that the genetic diversity of *D. angustifolia* was low in the population level. AMOVA was used to study genetic variation in *D. angustifolia*. At the species level, 95% of genetic variation came from among the population, and 5% from within the population (Table 3). AMOVA results showed significant genetic variation among populations (PhiPT = 0.946, *p* < 0.0001). Pairwise population AMOVA showed similar results (Table S5). We visualized patterns of genetic variation through principal coordinate analyses (PCoA) based on Nei’s genetic distance matrix (Figure 1a), and PCoA could explain 30.43% of genetic variation. PCoA analysis showed that all *D. angustifolia* populations can be divided into two large groups along the 1 axis of the principal coordinate, and P01–P09 is the first large group, of which P01–P05 and P06–P09 can be divided into two groups along the 2 axis of the principal coordinate. P10–P17 is the second large group, and P10–P13 and P14–P17 are further divided into two groups along the principal coordinate 2 axis. Based on Nei’s genetic distance, the unweighted pair group mean with arithmetical averages (UPGMA) clustering analysis was carried out on 17 populations of *D. angustifolia* (Figure S2a). The results showed that 17 populations of *D. angustifolia* were clearly separated into two clusters: the first cluster was separated in two subgroups, the first one included P04, P05, P06, P07, P03, P01, P02; the second one included P08, P09, P10, P11. The second cluster was also separated into two subgroups, the first one included P12, P13, P14, the second one included P17, P15, P16. PCoA and clustering analysis indicated that there were significant genetic variations in different populations.

### 2.2. DNA Methylation Level

**Figure 2:**
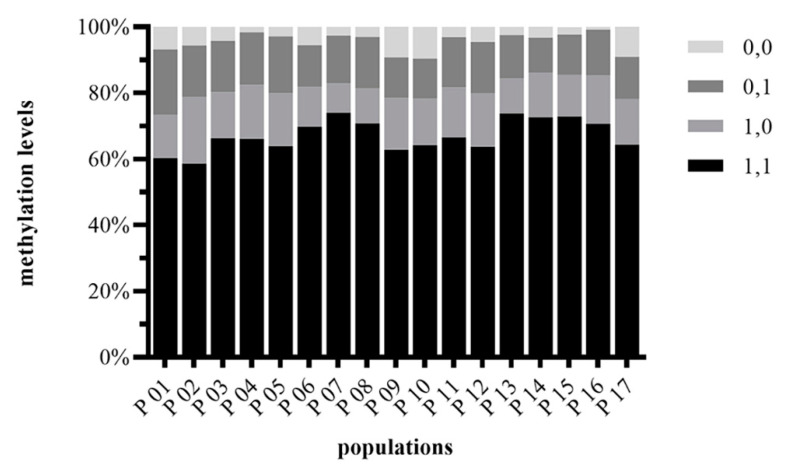
Relative DNA methylation levels in all populations of *D. angustifolia.* I—fragments present in both profiles (1,1), unmethylated state; II—fragments were present only with EcoRI/HpaII (1,0), hemi-methylation of external cytosine; III—fragments were present only with EcoRI/MspI (0,1), full-methylation of internal cytosine; IV—fragments were absent with both profile (0,0), uninformative site.

DNA methylation levels were significantly different in the 17 *D. angustifolia* populations tested. Among all populations, the percentage of unmethylated states was highest. In population P01, P03, P05, P06, P07, P08, P13, the level of total methylation was higher than that of semi-methylation, but in other populations, the level of total methylation was lower than that of semi-methylation (Figure 2). The full methylation levels were between 10.60% (P14) and 19.75% (P01), with an average of 14.35%; the semi-methylation levels were between 8.98% (P07) and 20.30% (P02), with an average of 14.00%; the total methylation levels were between 23.36% (P07) and 35.70% (P02), with an average of 28.35% (Table S3). The results showed that the level of DNA methylation of *D. angustifolia* was relatively high in different populations, and there were different methylation patterns among different populations.

### 2.3. Epigenetic Diversity and Structure

Using MSAP molecular marker technology, 10 pairs of selective amplification primers were used to analyze the epigenetic diversity and epigenetic structure of 17 natural *D. angustifolia* populations. A total of 2345 loci were detected, the Shannon’s information index (I) for each population ranged from 0.015 (P08) to 0.199 (P17), with an average of 0.054; the percentage of polymorphic loci (PPL) ranged from 2.87% (P08) to 37.95% (P17), with an average of 10.59% (Table 2). The results showed that the epigenetic diversity of *D. angustifolia* was low in the population level. AMOVA results showed that at the species level, 87% of epigenetic variation came from among population and 13% from within population (Table 2). AMOVA results showed significant epigenetic variation among populations (PhiPT = 0.866, *p* < 0.001). Pairwise population AMOVA showed the similar results (Table S5). We visualized patterns of epigenetic variation through PCoA based on Nei’s epigenetic distance matrix (Figure 1b), and PCoA could explain 17.02% of epigenetic variation. PCoA analysis showed that, unlike genetic variation, P05 was significantly separated from other populations, while P01, P02 and P03 were separated from other populations along the 1 axis of the principal coordinate. In addition, P04 was separated from the rest of the population along the 2 axis of the principal coordinate. Based on Nei’s epigenetic distance, UPGMA clustering analysis was carried out on 17 populations of *D. angustifolia*. The results showed that the 17 populations of D. angustifolia were clearly separated into two clusters (Figure S2b): the first cluster included P05 and the second cluster included all the other populations. PCoA and clustering analysis indicated that there was significant epigenetic variation in different populations.

### 2.4. Differences in Soil Properties

**Figure 3:**
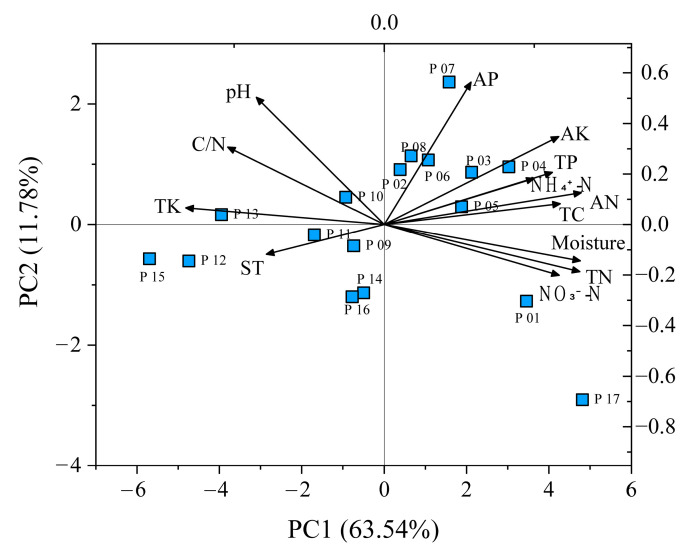
Principal component analysis (PCA) of soil properties in different *D. angustifolia* populations. Gray arrows indicate environmental factors, the red squares indicate *D. angustifolia* sampling plots. TC: total organic carbon, TN: total nitrogen, C/N: carbon–nitrogen ratio, NH_4_⁺-N: ammonium nitrogen, NO_3_^−^-N: nitrate nitrogen, TP: total phosphorus, TK: total potassium, AN: available nitrogen, AP: available phosphorus, AK: available potassium, Moisture: water content, ST: soil temperature.

Principal component analysis (PCA) was used to show the variation of soil properties in different sampling sites, and PCA explains 75.32% of the variation (Figure 3). PC1 (Principal component 1) and PC2 (Principal component 2) explain 63.54% and 11.78% of the variation, respectively. P09–P16 are obviously separated from other plots along the 1 axis of the principal component, which is mainly affected by the changes of soil temperature, total potassium, carbon–nitrogen ratio and pH, while other communities are mainly affected by the changes of soil moisture, total nitrogen, ammonium nitrogen, nitrate nitrogen, total organic carbon and total phosphorus. In addition, P17 was obviously separated from other communities along the 1 axis of the principal component. PCA results showed that there was obvious heterogeneity in the growth environment of *D. angustifolia* in different populations.

### 2.5. Environmental Drivers of Genetics and Epigenetics

**Figure 4:**
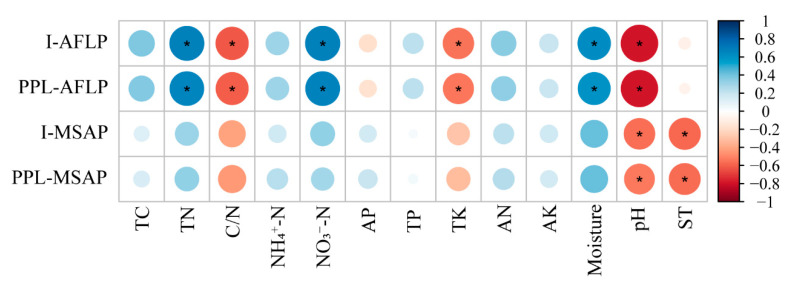
Correlation analysis of soil properties with genetic diversity and epigenetic diversity of *D. angustifolia*. TC: total organic carbon, TN: total nitrogen, C/N: carbon–nitrogen ratio, NH_4_⁺-N: ammonium nitrogen, NO_3_^−^-N: nitrate nitrogen, TP: total phosphorus, TK: total potassium, AN: available nitrogen, AP: available phosphorus, AK: available potassium, Moisture: water content, ST: soil temperature; I: Shannon’s information index; PPL: percentage of Polymorphic Loci. * Correlation is significant at the 0.05 level.

From the pairwise correlation analysis (Figure 4), soil TN, NO_3_^−^-N and moisture (soil total nitrogen, nitrate nitrogen and water content) were positively related to the genetic diversity of *D. angustifolia*; soil C/N, TK and pH (soil carbon–nitrogen ratio, total potassium and pH) were negatively related to the genetic diversity of *D. angustifolia* (*p* < 0.05, Figure 4). For epigenetic diversity, only soil pH and soil temperature were significantly negatively correlated with epigenetic diversity of *D. angustifolia* (*p* < 0.05, Figure 4, Figure S3a). We found that soil temperature decreased significantly with the increase in elevation (r = −0.5966, *p* = 0.0115). DNA methylation in plants is usually sensitive to the changes of environmental factors, and the changes of soil temperature had no significant effect on the levels of full methylation and semi-methylation (*p* > 0.05, Figure S3c,d). However, we found that soil temperature had a significant effect on the total methylation level of *D. angustifolia* (r = 0.5670, *p* = 0.0176, Figure S3b), that is, the decrease in soil temperature would significantly increase the total DNA methylation level of *D. angustifolia*.

Simple Mantel tests between genetic variation and epigenetic variation with geographical distance and environmental factors across all sites revealed that geographical distance (r = 0.647, *p* < 0.001) and environmental factors (r = 0.127, *p* = 0.041) were significantly correlated with epigenetic variation (Table 4). In addition, there was no significant correlations between epigenetic variation with genetic variation, geographical distance and environmental factors. After eliminating the effect of genetic variation, partial Mantel tests results showed that environmental factors and geographic distance were not significantly correlated with epigenetic variation across all sites. While eliminating the effect of epigenetic variation, environmental factors (r = 0.132, *p* = 0.033) and geographic distance (r = 0.649, *p* < 0.001) were significantly correlated with genetic variation across all sites. When we eliminated the effect of geographic distance or environmental factors, there was no significant correlation between genetic variation and epigenetic variation. In addition, when we eliminated the effect of geographic distance, environmental factors were not significantly correlated with genetic variation and epigenetic variation, but when we eliminated the effect of environmental distance, geographic distance was significantly correlated with genetic variation (r = 0.647, *p* < 0.001) and epigenetic variation (r = 0.251, *p* = 0.015) (Table 5).

## 3. Discussion

*D. angustifolia*, a local herbaceous plant in the low altitude area of the Changbai Mountains, has gradually expanded upward to the tundra belt with relatively high altitude in recent years due to natural disasters (typhoons) and air nitrogen deposition. The molecular mechanisms of genetics and epigenetics may provide the basis for the adaptation of *D. angustifolia* to the new environment. In order to understand how *D. angustifolia* adapts to the tundra environment through genetics and epigenetics, AFLP and MSAP analyses were carried out on 17 natural populations of *D. angustifolia* in an elevation gradient on the western slope of the Changbai Mountains, so as to deepen our understanding of the ecological adaptability of *D. angustifolia*.

### 3.1. Genetic Diversity and Structure

Consistent with previous research results on other clonal plants [32,38], the AFLP results showed that the genetic diversity of *D. angustifolia* was relatively low, and the average values of the Shannon information index (I) and percentage of polymorphic loci (PPL) in 17 populations were 0.035 and 6.79%, respectively (Table 2). According to the previous research of our research group, *D. angustifolia* has the characteristics of seed reproduction and rhizome asexual reproduction. In the process of upward expansion, sexual colonization was carried out at first, and then the ramets produced by clonal reproduction grew rapidly in the new habitat. In addition, although the offspring produced by sexual reproduction have higher genetic diversity, the efficiency of asexual reproduction is relatively high [39], so the expansion mode based on clonal reproduction is very important to the rapid occupation of the niche and the success of expansion. Due to the lack of meiosis mechanism, especially the basic stages such as hybridization and gene recombination, vegetative reproduction is expected to reduce genetic diversity. In the process of plant invasion, invasive plants, especially clonal plants, may maintain a low level of genetic diversity after gene drift during invasion [40,41], so genetic drift may be one reason for the low genetic diversity of the invasive population. In addition, studies on clonal plant species showed that ramets exhibit different phenotypes in different environments and thrive in different environments, leading to a serious reduction or lack of biological genetic diversity [32,42]. AMOVA results showed that there was very little genetic variation in *D. angustifolia*, and only 5% of the genetic variation came from within the population (Table 3), which may be related to the rapid occupation of the habitat by a large number of clones after expansion and colonization. Similar to the genetic variation of *D. angustifolia*, the results of studies on *A.donax* and *Alternanthera philoxeroides* showed that the genetic variation is very small within the scope of invasion [43,44,45]. In addition, the study on *Capsella rubella* showed that although the genetic variation is very low, plants can quickly produce phenotypic variation through the massive amplification of transposons, thus improving their adaptability [12]. PCoA (Figure 1a) and clustering analysis (Figure S2a) indicated that there were significant genetic variations in different populations. In addition, we found that the genetic variation of different populations is basically distributed along the elevation gradient, which may be due to the influence of geographic distance formed by the elevation gradient.

### 3.2. Epigenetic Diversity and Structure

The low genetic diversity of *D. angustifolia* does not affect the upward expansion of *D. angustifolia* into the tundra where the environment is more complex and changeable. There is now increasing evidence that heritable variation in ecologically related traits can be generated through a set of epigenetic mechanisms even in the absence of genetic variation [32]. In addition, studies have shown that epigenetic changes in natural populations can be independent of genetic variation, and in some cases, environment-induced epigenetic changes may be inherited by offspring [24,38].

Similar to the AFLP results, we found that at the population level, the epigenetic diversity of *D. angustifolia* was relatively low. The average values of the Shannon information index (I) and percentage of polymorphic loci (PPL) were 0.038 and 9.21%, respectively (Table 2). AMOVA results showed that there was significant epigenetic variation among populations (PhiPT = 0.929, *p* < 0.0001). A total of 87% of the epigenetic variation originated among populations, and 13% of the epigenetic variation originated within population (Table 3). Similar to previous studies of epigenetic variations in natural populations of other species [43,46], epigenetic variations within populations are higher than genetic variations within populations (MSAP: 13%, AFLP: 5%). A reasonable explanation is that since the spontaneous epigenetic mutation rate is higher than the genetic mutation rate, the epigenetic variation is usually larger [47], which may separate the genetic variation from the epigenetic variation. Another common explanation is that populations with limited genetic diversity, especially after genetic drift, can expand their niche through epigenetic variation, which may be sensitive to environmental stimuli [48]. PCoA (Figure 1b) and clustering analysis (Figure S2b) indicated that there were significant genetic variations in different populations. However, the degree of separation of epigenetic variation among different populations is not as obvious as genetic variation. This means that the effect of geographic distance on epigenetic variation is not obvious.

### 3.3. Environmental Drivers of Genetics and Epigenetics

Total nitrogen and nitrate nitrogen are positively correlated with genetic diversity of *D. angustifolia* (Figure 4), which indicates that nitrogen plays an important role in the expansion of *D. angustifolia*. Since the western slope faces the wind and increases the accumulation of nitrogen, the growing season is mostly westerly, which means that precipitation brings more atmospheric nitrogen deposition. Previous studies have suggested that nitrogen deposition is an important reason for the upward expansion of low-altitude plants [49]. Zong et al. also believed that adequate nitrogen supply is essential for the upward expansion of *D. angustifolia*, which enables it to reproduce and expand rapidly by increasing the number of tillers [36,50], which can provide conditions for the colonization of *D. angustifolia* in the tundra zone and increase the genetic diversity to some extent. There is a positive correlation between soil moisture content and genetic diversity (Figure 4). The elevation of the tundra zone is relatively high, and the temperature decreases with the increase in elevation, so low evaporation leads to higher soil moisture content. *D. angustifolia* is a hydro-mesophyte perennial herb, so sufficient water is beneficial to its establishment and growth. There was a significant negative correlation between pH and the genetic diversity of *D. angustifolia* (Figure 4), which meant that the increase in soil pH would reduce the genetic diversity of *D. angustifolia*. A study on the tree species *Beilschmiedia roxburghiana* also found similar results: as soil pH increased, the genetic diversity of trees decreased. One explanation for this is that with the increase in pH, the average niche width of species decreased, leading to the decrease in genetic diversity [51]. Therefore, we believe that lower soil pH may increase the niche width of *D. angustifolia* and thus improve its genetic diversity, resulting in its better adapting to the tundra environment. Combined with the results above, the higher water content, soil nitrogen content and lower soil pH in the tundra zone are conducive to the colonization of *D. angustifolia*, which will increase their genetic diversity to a certain extent, so as to better adapt to the environment in the tundra zone.

In plants, DNA methylation is involved in the response to environmental variations stimuli and/or stress. Changes in DNA methylation are associated with many biological functions and are considered as molecular tools for plants to adapt to different habitats and external stimuli [43,52]. Through MSAP molecular marker technology, we found that the total methylation level of the *D. angustifolia* genome was relatively high, with an average of 28.35% (Figure 2, Table S3), and the differences were significant among different populations. There was a significant correlation between DNA methylation level and soil temperature. Soil temperature was negatively correlated with the epigenetic diversity of *D. angustifolia* (Figure 4 and Figure S3a), and DNA methylation is more sensitive to environmental changes, which may be a molecular basis for the rapid adaptation of *D. angustifolia* to the changeable tundra environment when genetic diversity is limited in a short period of time. Previous studies have shown that epigenetic effects can be long-term or short-term involved in plant adaptation [32,53]. In populations, epigenetics can provide phenotypic flexibility beyond genetic constraints, allowing rapid responses to variable or unpredictable environments, potentially compensating for low genetic variability. Therefore, in the case of relatively low genetic diversity and genetic variation, *D. angustifolia* may adapt to the environments through epigenetic regulation.

Simple Mantel tests and partial Mantel tests showed that there was no significant relationship between genetic mechanisms and epigenetic mechanisms on the population level (Table 4 and Table 5). This is consistent with the results of previous studies on cloning intruder *Alternanthe raphiloxeroides* [38]. The results suggest that the genetic mechanism and the epigenetic mechanism may play an independent role in the adaptation process of *D. angustifolia.* There was a significant correlation between genetic variation and environmental factors, which indicates that environmental factors were an important source of genetic variation and suggests that *D. angustifolia.* is specifically adapted to particular environments. Consistent with the results of other plant species [23,54], there is a significant correlation between geographical distance and genetic variation. Combining the results of PCoA and clustering analysis, we believe that changes in elevation gradients have caused differences in the geographic distances of different populations, and geographic distance will affect the environmental factors of habitats, which in turn affect genetic variation.

In this study, we found that the genetic diversity of *D. angustifolia* was relatively low, and its adaptability could not be fully explained from the perspective of genetic diversity, but the soil properties in the tundra zone may be conducive to the colonization of *D. angustifolia*. In addition, our results suggest that epigenetic regulation, especially DNA methylation, can provide the basis for rapid adaptation to the environments. Our conclusion is only applicable to the methods used in this study, but this does not fully explain its adaptive mechanism. Therefore, carefully designed common garden studies are needed to partition the contributions of genetic and epigenetic variation to adaptive phenotypes.

## 4. Materials and Methods

### 4.1. Plant Materials

*Deyeuxia angustifolia* (Komarov) Y. L. Chang (*D. angustifolia*), a narrow-leaf small reed, is characterized as a hydro-mescophyte perennial herb, with life traits that include fast growth, clonal and sexual reproduction, a short juvenile period, high seed production and anemochory. In July 2017, 17 natural populations of *D. angustifolia* were selected from the alpine tundra on the western slope of the Changbai Mountains (Figure S1, Table 1), located in the Changbai Mountain National Nature Reserve (41°41′49″–42°25′18″ N, 127°42′55″–128°16′48″ E), Jilin province, China. The elevation of these populations ranged from 2041 to 2242 m. In each population, we selected 10 individuals for leaf sample collection. To ensure that the samples were not from the same physiological individual, we kept 1 m as a minimum distance between samples. Moreover, to avoid differences, the leaf samples used for molecular analysis were collected from the top two leaves of the plant. The collected leaves were quickly dried in silica gel for genomic DNA extraction.

### 4.2. Soil Sampling

At the same time of collecting plant samples, we randomly selected five plots in each population group and removed the surface plant samples and litter layers. Soil samples were collected with a soil drill (diameter of 5 cm), and all the soil was mixed evenly. About 500 g of soil was obtained at each site. All samples were collected on the same day. After collection, the samples placed in a cooler on ice were brought back to the laboratory for mixing, sifting and root removal. The collected soil samples were used for analysis of physical and chemical properties. Soil temperature was measured in situ using a datalogger (FOTEL, Shanghai, China).

### 4.3. Plant Genomic DNA Extraction

The total DNA of the plant leaves was extracted using a Plant Genomic DNA kit (Dingguo, Beijing, China), and the concentration and quality of the DNA were detected using a Nanodrop 2000 spectrophotometer (Thermo Scientific, Waltham, MA, USA), followed by verification by 1.2% agarose gel electrophoresis. The extracted DNA samples were stored in a refrigerator at −20 °C for later use.

### 4.4. AFLP Analysis

The amplified fragment length polymorphism (AFLP) fingerprinting followed the previous protocol of our research team with some modifications [55]. For restriction digestion and ligation, 10 μL volumes containing 60 ng genomic DNA were combined with 1.0 μL 10 × T4 DNA ligase buffer, 0.25 μL EcoRI adapter (20 μM), and 0.25 μL MseI adapter (20 μM), 5 U EcoRI (New England Biolabs, NEB, Ipswich, MA, USA), 5 U MseI (NEB), 0.125 μL T4 DNA ligase (NEB) and double-distilled water. The mixture was incubated at 37 °C for 8 h, 16 °C for 4 h, inactivated at 65 °C for 10 min and stored at −20 °C. For the preselective amplification (PCR1), 25 μL reaction system was combined with 2 μL digestion–ligation products, 0.25 μL of each pre-amplification primer, 12.5 μL 2 × EasyTaq PCR Supermix for PAGE (Transgen, Beijing, China) and 10 μL double-distilled water. The pre-amplification conditions were pre-denaturation at 94 °C (5 min) followed by 35 cycles of 94.0 °C (30 s), 56.0 °C (30 s) and 72.0 °C (80 s), a final extension at 72.0 °C (10 min) and storage at −20 °C. The PCR1 product was diluted by a ratio of 1:30. For the selective amplification (PCR2), 25 μL reaction system was combined with 2 μL PCR1 products, 1.0 μL of EcoRI and MseI primer (Table S1), 12.5 μL 2 × EasyTaq PCR Supermix for PAGE (Transgen, Beijing, China) and 8.5 μL double-distilled water. The selective amplification conditions were pre-denaturation at 95 °C (5 min) followed by 12 cycles of 94.0 °C (45 s), 65.0 °C (30 s) decreasing by 0.7 °C per cycle, and 72.0 °C (90 s), then followed by 23 cycles of 94.0 °C (45 s), 56.0 °C (30 s) and 72.0 °C (90 s), extension at 72.0 °C (10min) and storage at −20 °C. All the PCR reactions were performed in T100^TM^ Thermal Cycler (BIO-RAD, Hercules, CA, USA). Based on the number of amplified fragments, 10 DNA samples of each population and 10 selective primer combinations were chosen for the AFLP analyses (Table S1). The fragments were separated on 6% sequencing gels and silver stained. To test the repeatability of AFLP results, six individuals from each population were completely replicated starting from the restriction/ligation reaction of AFLP. For every polymorphic locus, each allele must exist in more than two individuals (>1% of all samples).

### 4.5. MSAP Analysis

The methylation sensitive amplified polymorphism (MSAP) technique, a modification of the amplified fragment length polymorphism (AFLP) markers which replaced the frequent cutter MseI with methylation-sensitive restriction enzymes HpaII (NEB) and MspI (NEB), was used to assess the epigenetic variation within and among populations. The MSAP protocol was performed with some modifications as the previous study described [56]. For AFLP analyses, ten DNA samples of each population multiplied by 10 selective primer combinations were chosen for the MSAP analyses (Table S2). The amplified fragments were separated according to the AFLP analyses protocol. The fragments were separated on 6% sequencing gels and silver stained. In order to reduce errors, the paired analyses with different enzymes of the same sample were run within the same gel. To assess the repeatability of our results, three individuals from each population were completely replicated starting from the restriction/ligation reaction of MSAP.

### 4.6. Soil Properties

Soil properties were determined within one month after sampling as previously described [37,57]. In brief, TC (total carbon) was determined by potassium dichromate-concentrated sulfuric acid oxidation method. TN (total nitrogen) was measured on an automatic kjeldahl nitrogen meter K1306 (Sonnen, Shanghai, China), AN (available nitrogen) was measured using the Illinois soil nitrogen test diffusion method, NH_4_⁺-N (ammonium nitrogen) and NO_3_^−^-N (nitrate nitrogen) were extracted with 2 M KCl, NH_4_⁺-N was determined by the indiophenol blue colorimetric method, while NO_3_^−^-N was determined by ultraviolet spectrophotometry. TP (total phosphorus) was determined colorimetrically using the molybdate method. AP (available phosphorus) was determined colorimetrically based on the Olsen method. TK (total potassium) was extracted by incubation with sodium hydroxide, and AK (available potassium) was extracted by incubation with 1.0 mol L^−1^ ammonium acetate for 0.5 h, followed by filtration. Soil pH was measured on a 1:5 (*w*/*v*) ratio in distilled water using a pH meter. Soil moisture was determined after drying at 105 °C for 24 h. The soil C/N ratio was calculated as the TC to TN ratio. Soil temperature of each population was measured in situ using a datalogger.

### 4.7. Statistical Analyses

The software Quantity One was used to identify and label distinct and independent bands of the electrophoresis. The AFLP and MSAP fragments (bands) were scored as presence (1) or absence (0) of DNA fragments (bands). All fragments from 100 to 600 bp were considered, and a data matrix of the AFLP and MSAP banding patterns of all populations was assembled for further analysis. Since the sensitivity of restriction enzymes HpaII and MspI to the methylation site is different, the HpaII only identifies cytosine lateral hemi-methylation (single chain), while the MspI only recognizes the inner cytosine full-methylation (double chain).The MSAP reactions can be divided into four distinct outcomes: I, fragments present in both profiles (1/1), unmethylated state; II, fragments present only with EcoRI/HpaII (1/0), hemi-methylation of external cytosine; III, fragments present only with EcoRI/MspI (0/1), full-methylation of internal cytosine; IV, fragments absent with both profiles (0/0), uninformative site. The first three results predict different methylation levels, while the last result may have different causes, including methylation mutations or DNA sequence mutations, and it is thus uninformative [58]. For further analyses, we transformed raw MSAP data matrix into a binary matrix as Schulz et al. described [58]. Methylation levels of DNA were obtained from as the percentages of the four results.

According to the binary AFLP and MSAP data matrix, the population genetics software GENALEX 6.5 [59] was used to analyze the main biodiversity indices within and between populations, including the Shannon’s information index (I), percentage of polymorphic loci (PPL); analysis of molecular variance (AMOVA) was used to study the distribution of genetic and epigenetic variation, with 9999 permutations. Pairwise population Φ_ST_ values were used to evaluate genetic and epigenetic differentiation among all groups based on GENALEX 6.5. Nei’s genetic distance and Nei’s epigenetic distance matrix were obtained based on GENALEX 6.5. Principal coordinate analysis (PCoA) was used to analyze genetic variation and epigenetic variation between populations. AMOVA and PCoA were also based on GENALEX 6.5 software. The MEGA X [60] software was used to conduct a cluster analysis on different populations using the unweighted pair group mean with arithmetical averages (UPGMA). Pearson correlation analysis between soil properties and genetic diversity index and epigenetic diversity index was performed in R (version 4.0.2) with the corrplot package. Mantel tests and partial Mantel tests were conducted with the vegan package in R, with 9999 permutations. Origin2018 was used for principal component analysis of soil properties. TC, TN, NH_4_⁺-N, NO_3_^−^-N, AP, pH, moisture and C/N ratio (means ± standard deviation) of each sample was performed with three technical repeats in the laboratory. For TP, TK, AK and AN, a single test of each sample was determined by the Public Technology Service Center of the Northeast Institute of Geography and Agroecology, Chinese Academy of Sciences with a 95% confidence level.

## 5. Conclusions

Finally, we found that the genetic diversity of *D. angustifolia* in the western slope of the Changbai Mountains was relatively low, which may be due to the short time of expansion into the tundra zone, and the clonal reproduction and genetic drift in the process of expansion. The epigenetic diversity is relatively low, but the level of DNA methylation is relatively high, and the main variations of the genetics and epigenetics of *D. angustifolia* were from different populations. This may be due to geographical isolation. Sufficient nitrogen, higher soil moisture and lower soil pH may provide a suitable environment for the colonization and expansion of *D. angustifolia* in tundra. Clonal reproduction and epigenetic regulation, especially DNA methylation, may be involved in the adaptation of *D. angustifolia* to different environments, which may be a molecular basis for rapid adaptation to tundra environment.

## Figures and Tables

**Table 1:** Basic information of *D. angustifolia* natural population sampling points.

	Elevation (m)	Latitude (°N)	Longitude (°E)	Nsp	ST (°C)
P01	2216	41.99077	128.01663	3	14.3
P02	2220	41.99142	128.01673	3	11.5
P03	2242	41.99280	128.01647	3	16.0
P04	2241	41.99272	128.01640	6	15.2
P05	2240	41.99268	128.01627	4	15.9
P06	2162	41.99052	128.01345	3	25.0
P07	2163	41.99053	128.01325	3	15.3
P08	2151	41.99018	128.01317	3	17.7
P09	2148	41.99065	128.01085	5	16.5
P10	2142	41.99053	128.01088	5	15.0
P11	2140	41.99050	128.01078	5	17.6
P12	2076	41.99030	128.00440	6	19.3
P13	2077	41.99033	128.00435	6	20.4
P14	2074	41.99020	128.00430	4	19.3
P15	2047	41.98882	128.00275	6	25.7
P16	2044	41.98880	128.00280	5	21.4
P17	2041	41.98855	128.00267	5	16.0

Nsp: number of species in sampling point (species richness index), ST: soil temperature.

**Table 2:** Genetic and epigenetic diversity of the natural population of *D. angustifolia.*

	Sample Size	I-AFLP	PPL-AFLP	I-MSAP	PPL-MSAP
P01	10	0.074	15.43%	0.073	18.98%
P02	10	0.025	4.90%	0.087	18.93%
P03	10	0.016	2.98%	0.051	11.98%
P04	10	0.051	9.23%	0.016	3.54%
P05	10	0.015	2.82%	0.018	4.61%
P06	10	0.034	6.19%	0.033	9.04%
P07	10	0.020	3.77%	0.029	7.68%
P08	10	0.009	1.75%	0.018	4.05%
P09	10	0.020	4.22%	0.061	14.80%
P10	10	0.023	4.50%	0.046	13.35%
P11	10	0.045	8.22%	0.044	9.08%
P12	10	0.026	5.29%	0.042	9.59%
P13	10	0.015	2.70%	0.011	2.26%
P14	10	0.014	2.48%	0.024	6.31%
P15	10	0.025	4.56%	0.020	4.73%
P 16	10	0.028	5.29%	0.006	1.36%
P17	10	0.164	31.14%	0.075	16.20%
Mean	10	0.035	6.79%	0.038	9.21%
SE	0.000	0.001	1.72%	0.001	1.38%

I = Shannon’s information index; PPL = Percentage of polymorphic loci.

**Table 3:** AMOVA results of amplified fragment length polymorphism (AFLP) data and methylation sensitive amplification polymorphism (MSAP) data of the natural population of *D. angustifolia.*

Source	df	Est. Var.	%	*p*-Value	Phi-Statistics
AMOVA results for AFLP data					
Among Pops	16	364.440	95%	0.0001	0.946
Within Pops	153	20.851	5%		
Total	169	385.291	100%		
AMOVA results for MSAP data					
Among Pops	16	214.356	87%	0.0001	0.866
Within Pops	153	33.065	13%		
Total	169	247.421	100%		

**Table 4:** Correlation coefficients using Simple Mantel tests across all sites.

	Gen	Epi
Epi	0.045	-
Geo	0.648 ***	0.137
Env	0.127 *	−0.099

Gen: Nei’s genetic distance, Epi: Nei’s epigenetic distance, Env: environment (soil) distance calculated with Euclidean, Geo: geographical distance. Bold values indicate significant differences (*** *p* < 0.001; * *p* < 0.05).

**Table 5:** Partial Mantel tests were conducted to compare the relative effects of environment and spatial distance on genetics and epigenetics.

	Controlling for Gen.dist		Controlling for Epi.dist		Controlling for Geo.dist			Controlling for Env.dist		
	Effect of Env.dist	Effect of Geo.dist	Effect of Env.dist	Effect of Geo.dist	Effect of Gen.dist	Effect of Epi.dist	Effect of Env.dist	Effect of Gen.dist	Effect of Epi.dist	Effect of Geo.dist
Gen.dist	-	-	0.132 *	0.649 ***	-	−0.058	−0.120	-	0.059	0.647 ***
Epi.dist	−0.106	0.142	-	-	−0.058	-	−0.154	0.059	-	0.181 *

Gen.dist is the Nei’s genetic distance matrix. Epi.dist is the Nei’s epigenetic distance matrix. Env.dist is the environmental heterogeneity matrix calculated with Euclidean. Geo.dist is the geographic distance matrix. Bold values indicate significant differences (*** *p* < 0.001; * *p* < 0.05).

## Data Availability

The data presented in this study are available on request from the corresponding author.

## Supplementary Materials

The following are available online at https://www.mdpi.com/2223-7747/10/2/291/s1, Figure S1: Maps of the seventeen *D. angustifolia* sampling sites on the western slope of the Changbai mountain. Jilin Province, China, Figure S2: UPGAM cluster analyses of different populations of *D. angustifolia* based on Nei’s genetic distance (a, AFLP data) and Nei’s epigenetic distance (b, MSAP data), Figure S3: Correlation analyses of soil temperature with Epigenetic Shannon’s Information Index (a), total methylation level(b), full methylation level(c) and semi-methylation level (d) of *D. angustifolia*, Table S1: Adaptor and primer sequence used in AFLP analysis, Table S2: Adaptor and primer sequence used in MSAP analysis, Table S3: Bands of different states and DNA methylation level of *D. angustifolia* from different populations, Table S4: Soil properties of different populations of *D. angustifolia*, Table S5: Pairwise population ΦST values of genetic (above) and epigenetic (below) variation.
