# Supplementary material for: Genetic and Epigenetic Changes during the Upward Expansion of Deyeuxia angustifolia Kom. in the Alpine Tundra of the Changbai Mountains, China
Source: Plants (Basel). 2021 Feb 3;10(2):291. doi: 10.3390/plants10020291 (PMC7913720; doi:10.3390/plants10020291)
Supplement: Supplementary file 1 [file plants-10-00291-s001.pdf]

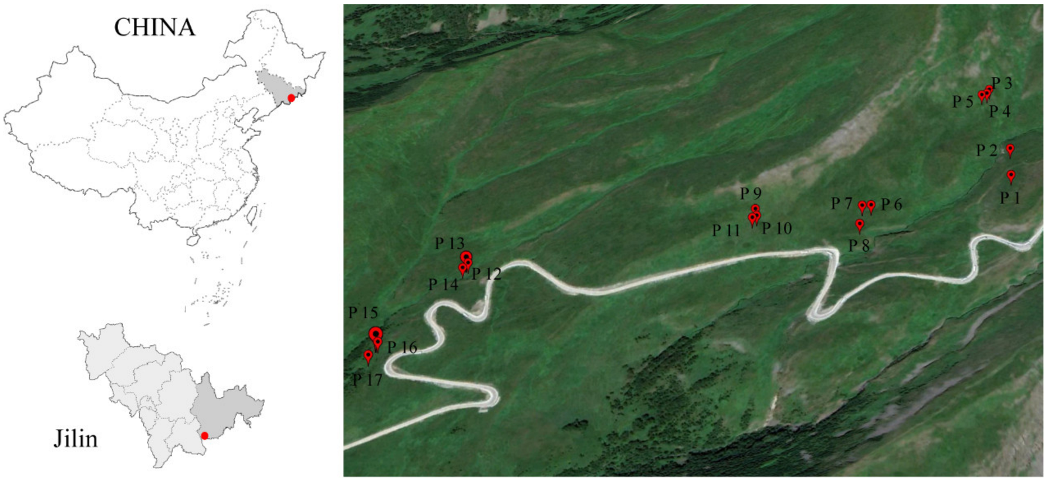

Figure S1. Maps of the seventeen *D. angustifolia* sampling sites on the western slope of the Changbai mountain. Jilin Province, China.

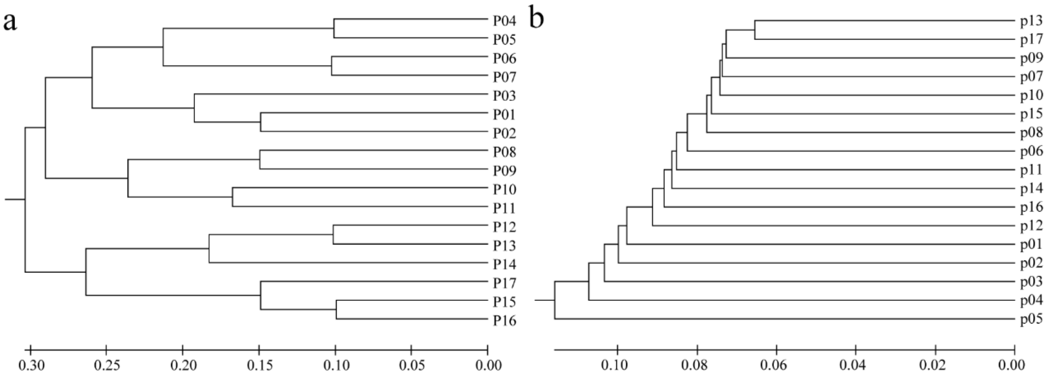

Figure S2. UPGAM cluster analysis of different populations of *D. angustifolia* based on nei genetic distance and epigenetic distance. a, AFLP data; b, MSAP data.

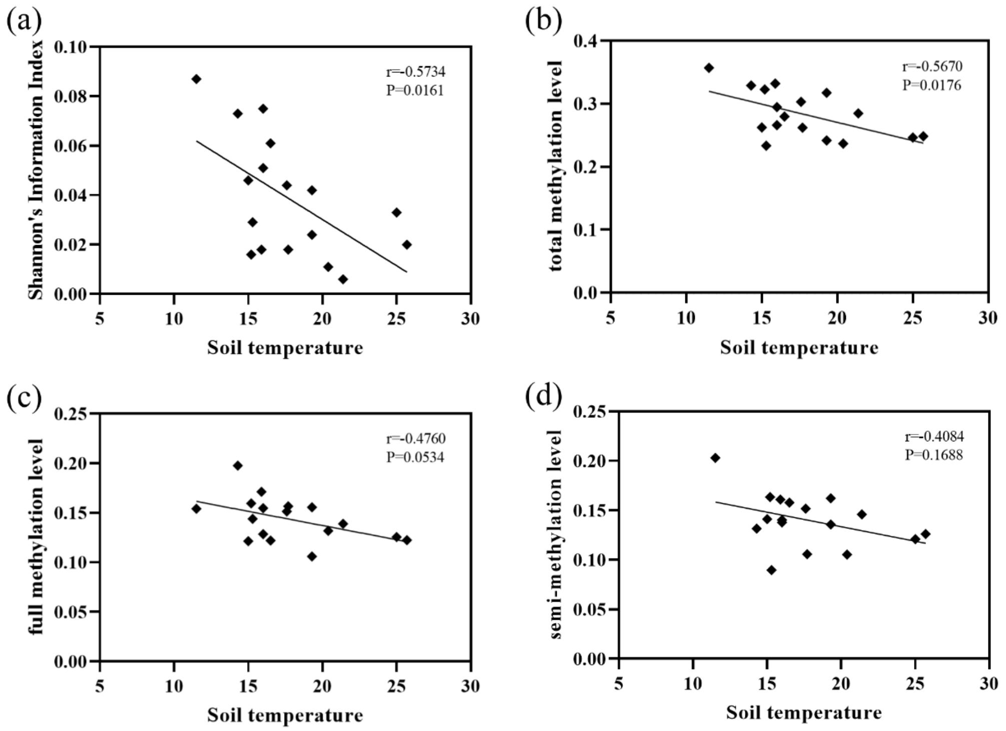

Figure S3. Correlation analysis of soil temperature with Epigenetic Shannon's Information Index (a), total methylation level(b), full methylation level(c) and semi-methylation level (d) of D. angustifolia

Table S1. Adaptor and primer sequence used in AFLP analysis

Primer	Sequence (from 5’ to 3’)
**Adapters**	
EcoRI_adapter top	CTCGTAGACTGCGTACC
EcoRI_adapter bottom	AATTGGTACGCAGTCTAC
MseI_adapter top	GACGATGAGTCCTGAG
MseI_adapter bottom	TACTCAGGACTCAT
**Preselective primers**	
EcoRI+A	GACTGCGTACCAATTCA
MseI+C	GATGAGTCCTGAGTAAC
**Selective primers**	
EcoRI+ACG[1]	GACTGCGTACCAATTCACG
EcoRI+AAC[2,3]	GACTGCGTACCAATTCAAC
EcoRI+ACC[4,5]	GACTGCGTACCAATTCACC
EcoRI+ACA[6]	GACTGCGTACCAATTCACA
EcoRI+ACT[7,8,9,10]	GACTGCGTACCAATTCACT
MseI+CTA[1,5,9]	GATGAGTCCTGAGTAACTA
MseI+CTT[2]	GATGAGTCCTGAGTAACTT
MseI+CTG[3,6]	GATGAGTCCTGAGTAACTG
MseI+CAA[4]	GATGAGTCCTGAGTAACAA
MseI+CAT[7]	GATGAGTCCTGAGTAACAT
MseI+CAC[8]	GATGAGTCCTGAGTAACAC
MseI+TCG[10]	GATGAGTCCTGAGTAACTC

Table S2. Adaptor and primer sequence used in MSAP analysis

Primer	Sequence (from 5’ to 3’)
**Adapters**	
EcoRI_adapter top	CTCGTAGACTGCGTACC
EcoRI_adapter bottom	AATTGGTACGCAGTC
HpaII/MspI_adapter top	GATCATGAGTCCTGCT
HpaII/MspI_adapter bottom	CGAGCAGGACTCATGA
**Preselective primers**	
EcoRI+A	GACTGCGTACCAATTCA
HpaII/MspI	ATCATGAGTCCTGCTCGG
**Selective primers**	
EcoRI+AAG[1,2,3]	GACTGCGTACCAATTCAAG
EcoRI+AGG[4,5]	GACTGCGTACCAATTCAGG
EcoRI+ACG[6,7,8]	GACTGCGTACCAATTCACG
EcoRI+AGC[9,10]	GACTGCGTACCAATTCAGC
HpaII/MspI+TCC[1,6]	ATCATGAGTCCTGCTCGGTCC
HpaII/MspI+TTA[2,5,7]	ATCATGAGTCCTGCTCGGTTA
HpaII/MspI+TGA[3]	ATCATGAGTCCTGCTCGGTGA
HpaII/MspI+TTG[4]	ATCATGAGTCCTGCTCGGTTG
HpaII/MspI+TAC[8,10]	ATCATGAGTCCTGCTCGGTAC
HpaII/MspI+TCG[9]	ATCATGAGTCCTGCTCGGTCG

Table S3. Bands of different states and DNA methylation level of *D. angustifolia* from different populations.

	1,0	0,1	1,1	0,0	tatol	full%	Hemi%	tatol%
P 01	159	239	728	82	1208	0.197517	0.131623	0.329139
P 02	234	178	675	65	1152	0.15408	0.202951	0.357031
P 03	162	179	766	49	1156	0.154598	0.140324	0.294922
P 04	174	170	705	17	1065	0.159437	0.16338	0.322817
P 05	199	212	790	36	1237	0.17114	0.161035	0.332175
P 06	128.6	133.6	742.6	59.2	1064	0.125564	0.120865	0.246429
P 07	94.6	151.6	780.3	27.5	1054	0.143833	0.089753	0.233586
P 08	95.6	141.6	640.6	27.2	905	0.156464	0.105635	0.262099
P 09	183.9	142.1	731.3	107.6	1164.9	0.121985	0.157868	0.279852
P 10	179.1	153.9	814	121	1268	0.121372	0.141246	0.262618
P 11	171.6	171.1	751.6	35.7	1130	0.151416	0.151858	0.303274
P 12	193	185	757.9	54.1	1190	0.155462	0.162185	0.317647
P 13	114.8	143.7	805.4	27.1	1091	0.131714	0.105225	0.236939
P 14	177.7	138.8	950.4	42.1	1309	0.106035	0.135752	0.241788
P 15	130.5	126.6	753.7	24	1034.8	0.122342	0.126111	0.248454
P 16	156.5	148.8	758.2	8.5	1072	0.138806	0.145989	0.284795
P 17	166.6	155.1	777.2	109.1	1208	0.128394	0.137914	0.266308

Table S4. Soil properties of different populations of *D. angustifolia*

	Elevation (m)	TC (g/Kg)	TN (g/Kg)	C/N	NH_4^+-N (mg/kg)	NO_3^--N(mg/kg)	TP (mg/kg)	TK (mg/kg)	AN (mg/kg)	AK (mg/kg)	AP (mg/kg)	Moisture (%)	pH
P 01	2216	137±8	13.49±0.64	10.15±0.33	88.03±1.65	33.89±0.81	887.54	14807.44	1071.84	133.08	87.49±3.74	68.49±0.13	4.29±0.02
P 02	2220	111.62±2.2	7.9±0.19	14.16±0.21	51.27±3.22	22.63±0.11	946.85	28216.59	966.00	148.83	108.82±4.06	52.36±0.2	4.56±0.02
P 03	2242	125.8±5.5	9.96±0.62	12.67±0.44	65.45±1.73	39.13±0.56	928.85	18798.38	1142.40	172.31	120.85±5.16	55.9±0.07	4.72±0.02
P 04	2241	133.95±5.82	11.43±0.45	11.73±0.26	87.63±2.29	44.78±4.28	985.78	18112.44	1125.60	194.77	104.56±6.71	53.95±0.12	4.85±0.01
P 05	2240	116.57±3.7	10.62±0.22	11±0.57	78.79±2.58	25.45±1.44	962.93	19780.42	1041.60	143.75	119.16±14.76	51.56±0.15	4.46±0.1
P 06	2162	119.08±4.84	9.45±0.32	12.66±0.9	68.66±2.62	27.21±1.34	1051.69	21169.87	960.96	170.52	136.35±10.43	47.7±0.13	4.58±0.03
P 07	2163	102.31±2.22	8.24±0.59	12.58±1.09	103.96±1.93	23.73±1.71	1027.13	20394.79	987.84	192.18	130.66±10.94	48.49±0.14	4.92±0.01
P 08	2151	125.29±4.49	8.93±0.2	14.6±0.76	40.33±0.92	23.58±1.88	913.35	21237.48	940.80	191.44	131.39±7.85	47.52±0.27	4.53±0.01
P 09	2148	76.1±0.65	6.9±0.24	11.06±0.42	72.61±2.22	20.95±1.09	856.90	22394.64	725.76	126.12	77.96±6.78	42.76±0.14	4.63±0.02
P 10	2142	81.9±0.45	6.86±0.29	11.99±0.57	72.49±2.25	14.57±0.25	776.32	23182.57	808.08	110.08	110.37±3.21	42.17±0.09	4.71±0
P 11	2140	58.65±0.78	5.01±0.04	11.71±0.26	57.18±1.24	24.14±0.24	834.16	24078.30	546.00	130.06	95.53±5.78	42.85±0.16	4.69±0.02
P 12	2076	63.54±1.27	4.75±0.13	13.41±0.56	36.85±1.38	13.82±1.25	540.57	37324.81	453.60	64.53	101.07±3.52	22±0.05	4.6±0.02
P 13	2077	82.58±1.61	5.02±0.07	16.47±0.35	54.78±1.93	14.59±1.42	738.57	32548.22	571.20	73.74	58.19±3.6	37.83±0.13	5.08±0.02
P 14	2074	96.37±1.4	8.32±0.28	11.61±0.5	55.54±0.61	26.86±0.38	953.98	24466.26	799.68	101.92	66.54±3.91	44.78±0.12	4.54±0.01
P 15	2047	74.75±0.36	4.41±0.01	16.96±0.1	34.76±1.14	13.66±1.79	575.95	35538.71	474.60	57.21	54.47±5.51	26.9±0.19	4.98±0.01
P 16	2044	100.57±3.28	8.25±0.33	12.2±0.1	61.58±1.65	18.34±0.09	970.48	24157.44	870.24	101.47	39.18±2.39	47.78±0.06	4.65±0.06
P 17	2041	124.71±7.18	14.36±0.2	8.68±0.41	73.42±1.02	54.62±3.78	945.10	11835.34	1108.80	172.85	78.98±2.95	70.28±0.05	3.7±0.02

TC: total organic carbon, TN: total nitrogen, C/N: carbon-nitrogen ratio, NH_4^+-N: ammonium nitrogen, NO_3^--N: nitrate nitrogen, TP: total phosphorus, TK: total potassium, AN: available nitrogen, AP: available phosphorus, AK: available potassium, Moisture: water content.

Table S5. Pairwise population Φ_{ST} values of genetic (above) and epigenetic (below) variation

	**Pop 1**	**Pop 2**	**Pop 3**	**Pop 4**	**Pop 5**	**Pop 6**	**Pop 7**	**Pop 8**	**Pop 9**	**Pop 10**	**Pop 11**	**Pop 12**	**Pop 13**	**Pop 14**	**Pop 15**	**Pop 16**	**Pop 17**
**Pop 1**	—	0.900**	0.932**	0.915**	0.946**	0.930**	0.940**	0.948**	0.939**	0.943**	0.919**	0.940**	0.947**	0.948**	0.942**	0.939**	0.843**
**Pop 2**	0.762**	—	0.954**	0.931**	0.971**	0.958**	0.968**	0.975**	0.966**	0.969**	0.944**	0.964**	0.972**	0.974**	0.968**	0.964**	0.869**
**Pop 3**	0.825**	0.800**	—	0.915**	0.973**	0.966**	0.975**	0.981**	0.972**	0.975**	0.950**	0.970**	0.978**	0.980**	0.973**	0.968**	0.876**
**Pop 4**	0.867**	0.845**	0.898**	—	0.890**	0.924**	0.947**	0.956**	0.948**	0.947**	0.924**	0.944**	0.952**	0.955**	0.946**	0.941**	0.845**
**Pop 5**	0.874**	0.851**	0.902**	0.954**	—	0.943**	0.970**	0.983**	0.974**	0.974**	0.952**	0.972**	0.980**	0.982**	0.976**	0.970**	0.872**
**Pop 6**	0.829**	0.812**	0.862**	0.919**	0.922**	—	0.918**	0.965**	0.962**	0.962**	0.941**	0.960**	0.968**	0.970**	0.964**	0.959**	0.861**
**Pop 7**	0.833**	0.818**	0.866**	0.926**	0.930**	0.882**	—	0.968**	0.966**	0.972**	0.949**	0.969**	0.977**	0.978**	0.974**	0.968**	0.873**
**Pop 8**	0.851**	0.831**	0.884**	0.942**	0.945**	0.899**	0.909**	—	0.960**	0.973**	0.956**	0.976**	0.983**	0.984**	0.979**	0.974**	0.875**
**Pop 9**	0.790**	0.770**	0.821**	0.872**	0.871**	0.828**	0.830**	0.842**	—	0.950**	0.939**	0.967**	0.974**	0.975**	0.970**	0.964**	0.863**
**Pop 10**	0.817**	0.798**	0.844**	0.897**	0.904**	0.854**	0.859**	0.878**	0.798**	—	0.917**	0.960**	0.974**	0.976**	0.970**	0.964**	0.859**
**Pop 11**	0.830**	0.815**	0.862**	0.916**	0.919**	0.879**	0.882**	0.900**	0.819**	0.852**	—	0.906**	0.938**	0.951**	0.946**	0.938**	0.838**
**Pop 12**	0.830**	0.813**	0.864**	0.909**	0.915**	0.876**	0.879**	0.897**	0.817**	0.848**	0.864**	—	0.930**	0.964**	0.966**	0.960**	0.861**
**Pop 13**	0.863**	0.835**	0.893**	0.953**	0.953**	0.916**	0.924**	0.939**	0.856**	0.890**	0.909**	0.902**	—	0.964**	0.969**	0.967**	0.867**
**Pop 14**	0.843**	0.818**	0.873**	0.929**	0.929**	0.891**	0.895**	0.914**	0.841**	0.866**	0.888**	0.884**	0.923**	—	0.963**	0.964**	0.865**
**Pop 15**	0.842**	0.819**	0.874**	0.937**	0.938**	0.894**	0.904**	0.922**	0.834**	0.866**	0.890**	0.887**	0.931**	0.908**	—	0.912**	0.809**
**Pop 16**	0.875**	0.848**	0.905**	0.960**	0.964**	0.928**	0.936**	0.953**	0.869**	0.906**	0.921**	0.916**	0.964**	0.937**	0.946**	—	0.735**
**Pop 17**	0.759**	0.744**	0.778**	0.833**	0.839**	0.792**	0.782**	0.797**	0.736**	0.749**	0.784**	0.780**	0.796**	0.791**	0.796**	0.826**	—

9,999 permutations, *p < 0.05, **p < 0.01.
